# Demonstration and imaging of cryogenic magneto-thermoelectric cooling in a van der Waals semimetal

**DOI:** 10.1038/s41567-024-02417-z

**Published:** 2024-03-08

**Authors:** T. Völkl, A. Aharon-Steinberg, T. Holder, E. Alpern, N. Banu, A. K. Pariari, Y. Myasoedov, M. E. Huber, M. Hücker, E. Zeldov

**Affiliations:** 1 Department of Condensed Matter Physics, Weizmann Institute of Science, Rehovot, Israel; 2 School of Physics and Astronomy, Tel Aviv University, Tel Aviv, Israel; 3 Departments of Physics and Electrical Engineering, University of Colorado Denver, Denver, CO USA

**Keywords:** Electronic properties and materials, Semiconductors

## Abstract

Attaining viable thermoelectric cooling at cryogenic temperatures is of considerable fundamental and technological interest for electronics and quantum materials applications. In-device temperature control can provide more efficient and precise thermal environment management compared with conventional global cooling. The application of a current and perpendicular magnetic field gives rise to cooling by generating electron–hole pairs on one side of the sample and to heating due to their recombination on the opposite side, which is known as the Ettingshausen effect. Here we develop nanoscale cryogenic imaging of the magneto-thermoelectric effect and demonstrate absolute cooling and an Ettingshausen effect in exfoliated WTe_2_ Weyl semimetal flakes at liquid He temperatures. In contrast to bulk materials, the cooling is non-monotonic with respect to the magnetic field and device size. Our model of magneto-thermoelectricity in mesoscopic semimetal devices shows that the cooling efficiency and the induced temperature profiles are governed by the interplay between sample geometry, electron–hole recombination length, magnetic field, and flake and substrate heat conductivities. The observations open the way for the direct integration of microscopic thermoelectric cooling and for temperature landscape engineering in van der Waals devices.

## Abstract (web summary)

Cooling efficiency in thermoelectric devices decreases considerably at lower temperatures. Now thermoelectric cooling at cryogenic temperatures is directly imaged in a van der Waals semimetal.

## Main

Thermoelectric effects have long been used for generating power, cooling electronic devices and measuring temperature where compact size, high stability, vibrationless operation without moving parts and high tunability are required^1,2^. Historically, research efforts have focused on elevated temperatures as the cooling efficiency decreases substantially at lower temperatures, such that it is eventually overshadowed by Joule heating for realistic current strengths. In recent years, the advent of high-purity single-crystal semimetals has led to increased interest in thermoelectric effects at cryogenic temperatures^3–12^.

The common approach to thermoelectric cooling is based on the Peltier effect^13^, in which a temperature gradient is attained along the current flow direction in structures composed of regions or materials with different Peltier coefficients, which, thus, carry different average amounts of heat per transferred unit charge. Peltier cooling of bulk materials down to liquid nitrogen temperatures has been realized in a number of studies^2,14^, with only one report of bulk cooling at liquid He temperatures^15^. By engineering microscopic devices that form energy gaps in the electron density of states, like superconducting junctions or quantum dots, the efficiency of Peltier cooling at very low temperatures can be enhanced substantially due to the preferential transmission of electrons at specific energies^16–18^.

**Fig. 1:**
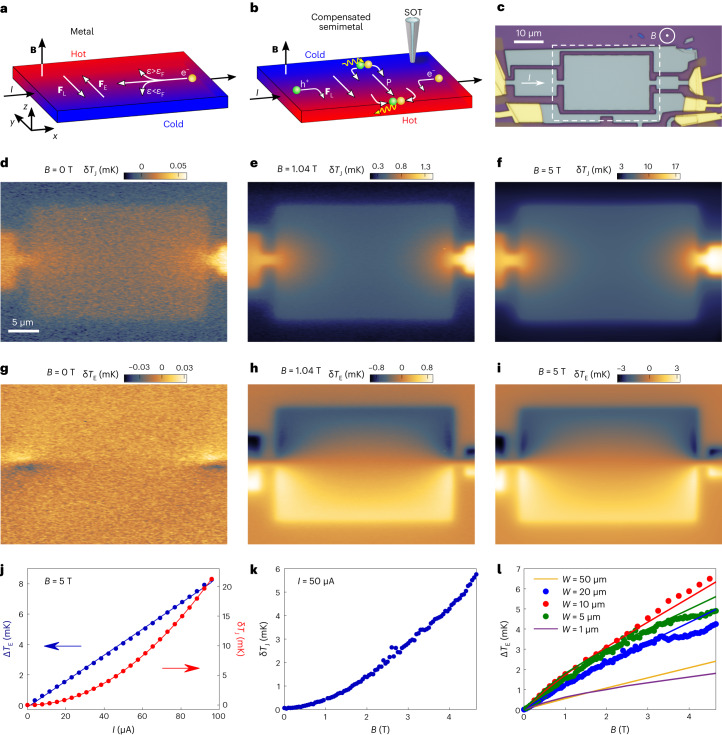
Thermal imaging of the Ettingshausen effect in a WTe_2_ flake at 4.3 K. **a**,**b**, Schematics of the Ettingshausen effect in metals (**a**) and in compensated semiconductors or semimetals (**b**) in the presence of a perpendicular magnetic field *B*. In a metal, the average Lorentz force **F**_L_ is counterbalanced by the transverse electrostatic force **F**_E_ of the Hall voltage. As the higher energy electrons usually have a lower drift velocity and, hence, lower **F**_L_, a small transverse thermoelectric effect develops. In semiconductors and semimetals, a much larger transverse temperature gradient of opposite sign is induced by electron–hole pair generation at the cold edge and recombination at the hot edge. **c**, Optical image of a WTe_2_ device showing three rectangular chambers (grey) of widths of *W* = 10, 20 and 5 µm along with surrounding additional patterns. A sinusoidal a.c. current *I* at frequency *f* is applied in series (white arrow) through the Au contacts (yellow). The dashed white rectangle marks the scan window in **d**–**i**. **d**–**f**, Temperature maps ${\updelta T}_\mathrm{J}(x,y)$ corresponding to Joule heating acquired at 2*f* with *I* = 5 µA at magnetic fields of *B* = 0 T (**d**), 1.04 T (**e**) and 5 T (**f**). **g**–**i**, Corresponding maps of Ettingshausen temperature component ${\updelta T}_\mathrm{E}(x,y)$ acquired simultaneously at *f* at magnetic fields of *B* = 0 T (**g**), 1.04 T (**h**) and 5 T (**i**). **j**, Left axis: temperature difference between the hot and cold edges Δ*T*_E_ (blue dots) versus *I* with a linear fit (blue line) at *B* = 5 T. Right axis: ${\updelta T}_\mathrm{J}({0,0})$ at the centre of the chamber versus *I* (red dots) and a parabolic fit (red line). **k**, Magnetic field dependence of δ*T*_J_ at *I* = 50 µA. **l**, Δ*T*_E_ versus *B* in the three adjacent chambers of *W* = 5, 10 and 20 µm at *I* = 50 µA (dots) showing nonlinear dependence on *B* and non-monotonic dependence on *W*. The solid curves are the corresponding Δ*T*_E_ from the numerical simulations. Source data

Here we address a very different and interesting magneto-electro-thermal mechanism, the Ettingshausen effect^19^, which has not been studied in microscopic devices previously^18^, and we present the observation of the Ettingshausen effect and Ettingshausen cooling at 4 K. The application of a charge current in the presence of a perpendicular magnetic field leads to a temperature gradient that is transverse to the current flow direction, rather than along it. In a metal, this effect is due to the energy dependence of the drift velocity of the charge carriers, which in the presence of magnetic field, deflects the high- and low-energy carriers to opposite sides of the sample, thus providing heating and cooling upon equilibration of the carriers with the lattice (Fig. 1a). The resulting current to temperature gradient conversion in this case is rather weak.

A much stronger effect can be attained in semiconductors and semimetals hosting both electrons and holes with approximately equal densities and mobilities^18,20^. Under such circumstances, the Hall voltage that usually develops across a device is diminished and the unbalanced Lorentz force drives both the electrons and holes in the same transverse direction (Fig. 1b). This leads to a non-equilibrium electron–hole pair accumulation on one side and depletion on the opposite side. As a result, recombination and generation of electron–hole pairs is increased, thus providing heating and cooling at opposing sides through equilibration with the lattice^19^. As such, heating and cooling are expected to be constrained to a small area within the recombination length *l*_R_ from the boundaries. Hence, a thermal measurement technique with spatial resolution on that length scale is required to fully elucidate the effect.

The search for suitable materials for thermoelectric cooling has focused predominantly on bulk systems, leaving the unrecognized potential of mesoscopic devices for integrated cooling largely unexplored^18^. Employing van der Waals (vdW) semimetals as thermoelectric elements^21–26^ is especially promising since they can be readily integrated into stacks of atomic layers and moiré heterostructures^27,28^. Herein, different materials, such as insulating, semiconducting, superconducting or magnetic layers, in the heterostructure can fulfil different functionalities. Accordingly, we propose the use of a vdW semimetal as an active thermoelectric element for in-device cooling or for engineering a controllable temperature gradient within a device. A particularly promising vdW material for thermoelectric cooling is the Weyl semimetal WTe_2_ because of its very high charge carrier mobility and near compensation of electron and hole densities^29–31^. In its bulk form, it has already been shown to display a large Nernst effect^32^ and ultrahigh Ettingshausen effect at temperatures above 20 K (ref. ^12^).

Thermoelectric mechanisms in which out-of-equilibrium voltages or currents are generated by temperature gradients, like the Seebeck and Nernst effects, have been investigated extensively in microscopic vdW devices. These studies are typically performed by measuring voltages across microscopic contacts induced by global temperature gradients^21–26,33–35^ or by local heating using, for example, a scanning focused laser beam^36–45^. In contrast, electro-thermal processes, like the Peltier and Ettingshausen effects, for which temperature gradients need to be measured or imaged in response to applied currents, are much more challenging to investigate in microscopic devices and have, thus far, been restricted to above liquid nitrogen temperatures^46,47^. In this work, we provide thermal imaging of magneto-electro-thermal cooling at liquid He temperatures in exfoliated flakes of the transition metal dichalcogenide WTe_2_, revealing an ultrahigh Ettingshausen effect and the underlying mesoscopic mechanisms that have been inaccessible experimentally hitherto.

## Cryogenic thermal imaging

A superconducting quantum interference device on a tip (SQUID-on-tip, SOT)^48^ made of MoRe (ref. ^49^) with a diameter of 110 nm was scanned above the sample surface at a height *h* ≈ 80 nm in an He exchange gas atmosphere. It was employed as a nanothermometer^50^ with 2.6 µK Hz^−^^1/2^ sensitivity at *T*_0_ = 4.3 K, operating in an applied out-of-plane magnetic field of up to *B* = 5 T (Methods), as shown schematically in Fig. 1b. A high-quality WTe_2_ single crystal^31^ was exfoliated to produce a *d* = 269 nm thick flake and patterned by reactive ion etching (Methods) into a number of rectangular chambers with different widths *W* (Fig. 1c). To circumvent the 1/*f* noise of the SOT, a sine or square-wave a.c. current at frequency *f* = 85.37 Hz and variable rms amplitude *I* was applied to the three chambers, which were connected in series by narrow constrictions (Fig. 1c). The resulting a.c. change in the local temperature δ*T*(*x*,*y*) relative to the base temperature *T*_0_ was imaged by the scanning SOT.

## Imaging the Ettingshausen effect in WTe_2_

The current-induced change in the local temperature δ*T* has two contributions, one arising from Joule heating δ*T*_J_ and the other from the Ettingshausen effect δ*T*_E_. By applying a sine-wave a.c. current through the sample, we can image these two contributions independently as follows. Since the Ettingshausen effect is linear in *I*, δ*T*_E_ is a sine-wave temperature modulation at the frequency *f* of the applied current. We, thus, detect this contribution by lock-in measurement at the fundamental frequency *f*. In contrast, Joule heating is quadratic in *I* and, therefore, results in d.c. heating superimposed by an a.c. temperature modulation at frequency 2*f* (Methods). We image this a.c. δ*T*_J_ modulation by lock-in detection at the second harmonic.

Figure 1d–f shows the Joule heating $\updelta {T}_{{\rm{J}}}(x,y)$ induced in the largest chamber (dashed rectangle in Fig. 1c) at *B* = 0, 1.04 and 5 T, respectively, at *I* = 50 μA (thermal images of the two smaller chambers of the device are shown in Extended Data Fig. 3). δ*T*_J_ was rather uniform in most of the chamber area with intense hot spots at the constrictions where the current density is high. Figure 1j shows that δ*T*_J_ in the chamber grew quadratically with *I*, as expected for Joule heating. δ*T*_J_ increased by over two orders of magnitude with the field, growing approximately quadratically with *B*, as shown in Fig. 1k. This is the consequence of the very large magnetoresistance ${\rho }_{{xx}}(B)\propto {B}^{2}$ in high-purity WTe_2_, as reported previously^29–32,51^.

The Ettingshausen temperature distribution $\updelta {T}_{{\mathrm{E}}(x,y)}$ is shown in Fig. 1g–i. At zero field, *δT*_E_ = 0 as expected (the weak signal in the constrictions is an artefact; Methods). Upon increasing the field, a large temperature gradient developed transverse to the current and magnetic field directions. The temperature difference ${\Delta}{T}_{\mathrm{E}}={\updelta}{T}_{\mathrm{E}}\left(0,-{W}/{2}\right)-{\updelta}{T}_{\mathrm{E}}\left(0,{W}/{2}\right)$ between the hot (bottom) and the cold (top) edges at $y=\mp W/2$ across the sample centre (*x* = 0) grew linearly with *I* (Fig. 1j), as expected for the Ettingshausen effect, ${\Delta T}_\mathrm{E}={P}_\mathrm{E}{IB}/W$, where *P*_E_ is the Ettingshausen coefficient. For *B* = 5 T and *W* = 20 μm, we found an ultrahigh Ettingshausen signal ${\Delta}{T}_{\mathrm{E}}/(JW)=2.2\times {10}^{-5}\,{\mathrm{K}}\,{\mathrm{A}}^{-1}\,{\mathrm{m}}$(*J* is the current density) comparable to the previously reported bulk values^12^ but at a much lower temperature. However, in contrast to the standard bulk behaviour, we found that Δ*T*_E_ is not linear in *B*, as shown in Fig. 1l. Even more surprisingly, investigating chambers with different widths revealed a non-monotonic *W* dependence, with the chamber with *W* = 10 μm showing a larger Δ*T*_E_ than those with *W* = 20 μm or *W* = 5 μm (Fig. 1l). Moreover, Fig. 1h,i shows a non-trivial spatial distribution of ${\updelta}{T}_{\mathrm{E}}(x,y)$ with enhanced heating and cooling signals on the left and right edges near the constrictions. These features were also observed in additional samples, as shown in Extended Data Figs. 2–4.

## Absolute cooling

The linear increase of the Ettingshausen temperature difference Δ*T*_E_ with current does not necessarily mean that the temperature of the cold edge of the sample decreases monotonically with *I* or that real cooling has been achieved at the cold edge. This is because the Joule heating usually has a dominant contribution. The first and second harmonic a.c. temperature variations in response to a sine-wave a.c. current presented in Fig. 1 allowed us to distinguish between the Ettingshausen and Joule terms, but these terms do not include the d.c. Joule heating component (Methods). To map the actual map of excess temperature, we modulated the applied current between zero and *I* by applying a unipolar square wave. In this case, the measured a.c. δ*T*(*x*, *y*) provides an image of the total current-induced excess temperature, which is the difference between the local temperature $T(x,y)={T}_{0}+{{\updelta}}T(x,y)$ in the presence of the current and the base temperature *T*_0_ in its absence. Positive δ*T* represents heating whereas negative δ*T* is cooling to below *T*_0_.

**Fig. 2:**
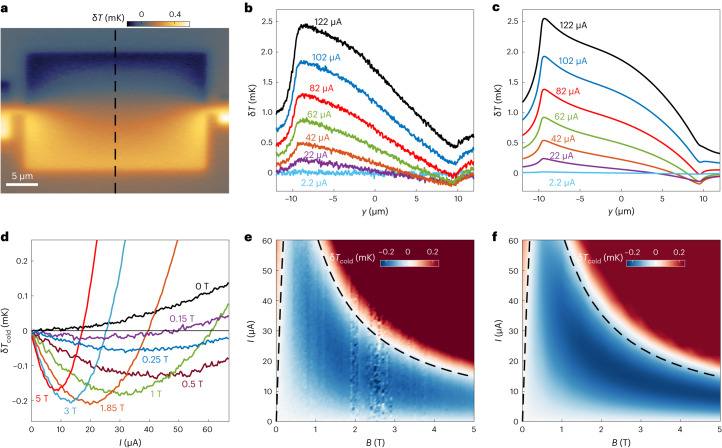
Absolute cooling. **a**, Map of the current-induced local excess temperature $\updelta T(x,y)$ relative to the base temperature *T*_0_ *=* 4.3 K, generated by a unipolar square-wave current excitation of *I* = 25 µA in the WTe_2_ device at *B* = 0.64 T. Regions with negative *δT* are cooled by the current to below *T*_0_. **b**, Profiles of excess temperature $\updelta T(0,y)$ along the black dashed line in **a** at various applied currents. **c**, Simulated temperature profiles $\updelta T(0,y)$. **d**, Excess temperature at the cold edge of the chamber $\updelta {T}_{\mathrm{cold}}$ versus *I* at different magnetic fields showing absolute cooling $\updelta {T}_{\mathrm{cold}} < 0$. **e**, Measured ${\updelta T}_{\mathrm{cold}}(I,B)$ diagram showing the parameter space of absolute cooling (blue). **f**, Simulated ${\updelta T}_{\mathrm{cold}}(I,B)$ diagram. The colour in the high-temperature regions (red) in **e** and **f** is saturated for clarity. The dashed lines in **e** and **f** show the fit of the current ${I}_{0}\left(B\right)$ from equation (6) below which absolute cooling occurs. Source data

Figure 2a shows the resulting temperature map δ*T*(*x*, *y*) in the *W* = 20 µm chamber at *B* = 0.64 T and *I* = 25 µA. δ*T*(*x*, *y*) along the top edge of the sample is negative. That is, the local temperature in the presence of the current is below *T*_0_, revealing current-induced absolute cooling. Even stronger cooling is observed in the top two corners of the chamber. To the best of our knowledge, this is the first observation of Ettingshausen cooling at liquid He temperatures.

Next, we explored the current and field dependence of the cooling mechanism. Figure 2b shows the temperature profile δ*T*(0, *y*) across the sample centre (dashed line in Fig. 2a) at *B* = 0.64 T and various applied currents *I*. The temperature at the hot edge ($y=-W/2$) increased monotonically with the current, and the temperature difference Δ*T*_E_ between the hot and cold edges increased linearly with *I* (Fig. 1j). Yet, the temperature at the cold edge ($y=W/2$) showed non-monotonic behaviour due to competition between the linear Ettingshausen cooling and the quadratic Joule heating. As a result, absolute cooling (δ*T* < 0) was achieved only at low currents. Note that at any current, the temperature at the cold edge was lower than the temperature at the hot edge or in the sample centre. That is, the relative Ettingshausen cooling always occurred, but at high currents it could not overcome the Joule heating to attain absolute cooling.

The cooling mechanism was also non-monotonic in magnetic field. Figure 2d shows $\updelta {T}_{\mathrm{cold}}=\updelta T(0,W/2)$ versus *I* at different *B*. At *B* = 0, the excess temperature was always positive, increasing monotonically with *I*. At finite fields, δ*T*_cold_ was negative at low currents, initially showing a negative slope with *I*, which increased monotonically with *B*. The current at which the maximal negative temperature ${\updelta T}_{\mathrm{cold}}^{\max }(B)$ is attained decreased monotonically with *B*, but $\updelta {T}_{\,{\mathrm{cold}}}^{\,\max }\left(B\right)$ has a non-monotonic *B* dependence with maximal negative ${\updelta T}_{\mathrm{cold}}^{\max }$ attained at *B* ≈ 2 T. Figure 2e shows the full map of $\updelta {T}_{\mathrm{cold}}(B,I\;)$, revealing a wide range of fields and currents at which negative excess temperatures can be achieved (blue).

## Theory of Ettingshausen effect in mesoscopic devices

The mechanism underlying the large Ettingshausen effect in WTe_2_ stems from the near compensation of electron and hole densities, ${n}_\mathrm{e}\approx{n}_\mathrm{h}=n$, and their mobilities, ${\mu}_\mathrm{e}\approx{\mu}_\mathrm{h}=\mu$. In bulk devices, the induced transverse temperature difference ${\Delta T}_\mathrm{E}={P}_\mathrm{E}{IB}/W$ is proportional to *B*/*W* (refs. ^12,19,20^). This relation does not hold in mesoscopic devices because of the competition between different microscopic length scales and due to the inherently non-uniform current distribution. Consider a bulk sample with uniform applied longitudinal electric field ${\mathbf{E}}={E}_{x}\hat{x}$. At *B* = 0 T, the resulting current density **J** comprises counterflowing particle flux densities of electrons **j**_e_ and holes **j**_h_, ${\mathbf{J}}=e({{\mathbf{j}}}_{\mathrm{h}}-{{\mathbf{j}}}_{\mathrm{e}})$, where *e* is the elementary charge. An out-of-plane magnetic field ${\mathbf{B}}=B\hat{z}$ gives rise to a transverse Lorentz force **F**_L_, which for a single-charge-type conductor is counterbalanced by the electrostatic force of the induced Hall voltage **F**_E_, resulting in the absence of magnetoresistance (Fig. 1a). In a compensated semimetal, in contrast, the Hall voltage vanishes and **F**_L_, which scales as *μB*, drives both the electrons and holes in the same transverse direction (Fig. 1b). This transverse flow of quasiparticles creates the quasiparticle current density ${\mathbf{P}}=e({{\mathbf{j}}}_{\mathrm{h}}+{{\mathbf{j}}}_{\mathrm{e}})$, which in turn causes a longitudinal Lorentz force that counteracts the force of the applied electric field, resulting in bulk magnetoresistance that scales as (*μB*)^2^, ${\rho }_{{xx}}\cong {{\rho }_{0}(1+\mu }^{2}{B}^{2})$, where *ρ*_0_ is the zero-field resistivity^29–32,51^.

**Fig. 3:**
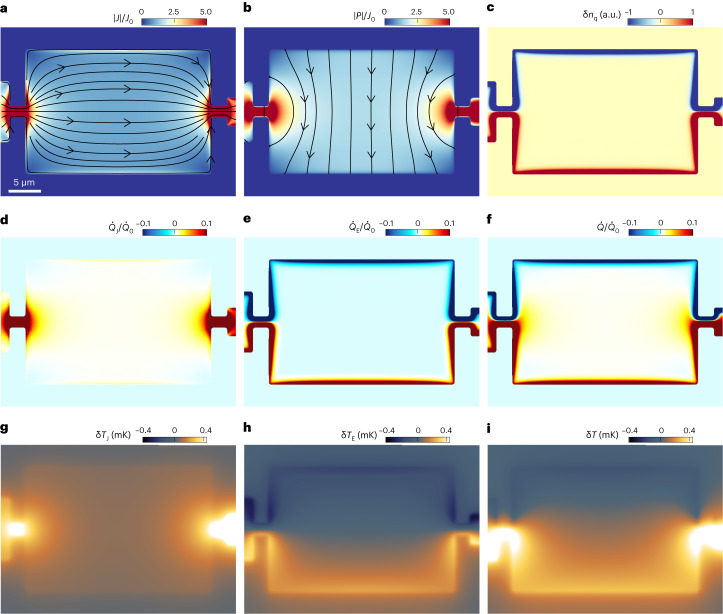
Numerical simulations of the magneto-thermoelectric response. **a**, Normalized current density distribution $\left|{{{\mathbf{J}}}}(x,y)\right|$ and streamlines at *B* = 0.64 T and *I* = 25 μA (*J*_0_ = *I*/*W*). **b**, Quasiparticle current density $\left|{{{\mathbf{P}}}}(x,y)\right|$ with streamlines. **c**, Out-of-equilibrium quasiparticle density $\updelta {n}_\mathrm{q}(x,y)$. The colours are saturated for clarity. **d**, Power density generated by Joule heating ${\dot{Q}}_\mathrm{J}(x,y)$. **e**, Power density ${\dot{Q}}_\mathrm{E}(x,y)$ produced by generation and recombination of quasiparticles due to the Ettingshausen effect. **f**, Total power density $\dot{Q}={\dot{Q}}_\mathrm{J}+{\dot{Q}}_\mathrm{E}$. **g**, Excess temperature distribution δ*T*_J_ caused by Joule heating. **h**, Excess temperature distribution δ*T*_E_ caused by the Ettingshausen effect. **i**, Total excess temperature $\updelta T(x,y)$. See Methods for simulation parameters.

The edges play a crucial role in a microscopic device. In particular, the boundary conditions preclude a transverse particle flow at the sample edges. This implies that the magnetoresistance, which is caused by the transverse flow, is suppressed near the boundaries. A recent theoretical study^52,53^ showed that the resulting current distribution across the width $-W/2\le y\le W/2$ of a long narrow strip is given by

1 $${J}_{x}\left(\,y\right)=\frac{{E}_{x}}{{{\rho }_{0}}(1+{\mu}^{2}{B}^{2})}\left[1+{\mu }^{2}{B}^{2}\frac{\cosh \left(2y/l_{\mathrm{R}}\right)}{\cosh \left(W/l_{\mathrm{R}}\right)}\right],$$

where ${l}_\mathrm{R}={l}_\mathrm{R}^{0}/\sqrt{1+{\mu }^{2}{B}^{2}}$ and ${l}_\mathrm{R}^{0}$ is the electron–hole recombination length at zero field. For ${l}_\mathrm{R}\ll W$, equation (1) shows that in the centre of the strip, the current density is suppressed by the magnetoresistance just like in the bulk limit, ${J}_{x}(\left|\,y\right|\ll W\;)=\frac{{E}_{x}}{{\rho }_{0}\left(1+{\mu }^{2}{B}^{2}\right)}$. Remarkably, along the edges, the current experiences no magnetoresistance, ${J}_{x}\left(\left|\,y\right|={W}/{2}\right)={{E}_{x}}/{{\rho }_{0}}$, remaining at its zero-field value. As a result, the current distribution peaks along the edges within narrow channels with a characteristic width of *l*_R_ (narrow bright slivers along the top and bottom edges in Fig. 3a).

In contrast to the conservation of charge current $\nabla \cdot {{{\mathbf{J}}}}=0$, the quasiparticle current **P** is not conserved because electron–hole pairs can be generated and recombined. Hence $\nabla \cdot {{{\mathbf{P}}}}=-\updelta {n}_\mathrm{q}/{\tau }_\mathrm{R}$, where $\updelta {n}_\mathrm{q}=\updelta {n}_\mathrm{e}+\updelta {n}_\mathrm{h}$ is the quasiparticle excess density, δ*n*_e_ and δ*n*_h_ are the out-of-equilibrium electron and hole densities, and *τ*_R_ is the electron–hole recombination time. In the narrow strip geometry, the resulting quasiparticle current and density distributions are given by

2 $${P}_{y}\left(\,y\right)=-\frac{\mu B{E}_{x}}{{\rho }_{0}\left(1+{\mu }^{2}{B}^{2}\right)}\left[1-\frac{\cosh \left({2y}/{{l}_\mathrm{R}}\right)}{\cosh \left({W}/{{l}_\mathrm{R}}\right)}\right].$$

3 $$\updelta {n}_\mathrm{q}(\,y)=-\frac{2\mu B{E}_{x}}{{\rho }_{0}\left(1+{\mu }^{2}{B}^{2}\right)}\frac{{\tau }_\mathrm{R}}{{l}_\mathrm{R}}\frac{\sinh \left({2y}/{{l}_\mathrm{R}}\right)}{\cosh \left({W}/{{l}_\mathrm{R}}\right)}.$$

In the central part of the sample, δ*n*_q_ is small because the quasiparticle thermal generation and recombination rates are approximately balanced, like in thermal equilibrium, and *P*_*y*_ is essentially constant, as shown in Fig. 3b,c. The transverse *P*_*y*_ driven by the Lorentz force gives rise to a sharp accumulation (depletion) of the quasiparticles within *l*_R_ from the bottom (top) edge. As a result, the electron–hole pair recombination rate is enhanced (suppressed) by $\updelta {n}_\mathrm{q}/{\tau }_\mathrm{R}$ at the bottom (top) edge, giving rise to heating by phonon emission (cooling by phonon absorption).

The thermoelectric effect can, thus, be derived by considering the two heat-generating terms $\dot{Q}={\dot{Q}}_\mathrm{J}+{\dot{Q}}_\mathrm{E}$, where ${\dot{Q}}_\mathrm{J}={{{\mathbf{J}}}}\cdot {{{\mathbf{E}}}}$ is the Joule term, ${\dot{Q}}_\mathrm{E}={\epsilon }_\mathrm{p}\updelta {n}_\mathrm{q}/{\tau }_\mathrm{R}=-{\epsilon }_\mathrm{p}\nabla \cdot {{{\mathbf{P}}}}$ is the Ettingshausen contribution and *ϵ*_p_ is the average phonon energy released (absorbed) by recombination (generation) of an electron–hole pair:

4 $${\dot{Q}}_\mathrm{J}(\,y)=\frac{{E}_{x}^{2}}{{\rho }_{0}\left(1+{\mu }^{2}{B}^{2}\right)}\left[1+{\mu }^{2}{B}^{2}\frac{\cosh \left({2y}/{{l}_\mathrm{R}}\right)}{\cosh \left({W}/{{l}_\mathrm{R}}\right)}\right],$$

5 $${\dot{Q}}_\mathrm{E}(\,y)=-\frac{2{\epsilon}_\mathrm{p}\mu B{E}_{x}}{e{\rho }_{0}{l}_\mathrm{R}^{0}\sqrt{1+{\mu }^{2}{B}^{2}}}\frac{\sinh ({2y}/{{l}_\mathrm{R}})}{\cosh({W}/{{l}_\mathrm{R}})}.$$

As expected, ${\dot{Q}}_\mathrm{J}$ is symmetric in *y* and quadratic in *E*_*x*_ and, hence, appears in the second harmonic for the a.c. current, whereas ${\dot{Q}}_\mathrm{E}$ is antisymmetric in *y* and linear in *E*_*x*_, consistent with the experimental results in Fig. 1.

To attain a better understanding of the thermoelectricity in mesoscopic systems, we ran three-dimensional (3D) finite-element numerical simulations for our sample geometry (Methods). Figure 3a shows that a high current density **J** is present in the constrictions, whereas in the rectangular chamber, the current density peaks along the sample edges where the magnetoresistance is suppressed. The corresponding Joule dissipation ${\dot{Q}}_\mathrm{J}$ is highest in the constrictions (Fig. 3d) and is enhanced in the narrow slivers along the top and bottom edges in the chamber. The quasiparticle current density ${{{\mathbf{P}}}}(x,y)$ in Fig. 3b also shows a large enhancement in the constrictions. In the rectangular chamber, **P** flows mostly transverse to **J** and is largest in the central area. Note that |${{{\mathbf{P}}}}\approx\mu B\mathbf{J}$| is substantially larger than |${{{\mathbf{J}}}}$| for the presented case of *μB* = 1.6, which corresponds to our experimental values at *B* = 0.64 T. The excess quasiparticle density $\updelta {n}_\mathrm{q}(x,y)$ sharply peaks along the sample boundaries, including the left and right edges (Fig. 3c). As a result, the Ettingshausen dissipation ${\dot{Q}}_\mathrm{E}$ (Fig. 3e) shows pronounced cooling along the edges in the top half of the sample and heating along the bottom-half edges. Depending on the parameters, the total dissipation $\dot{Q}={\dot{Q}}_\mathrm{J}+{\dot{Q}}_\mathrm{E}$ can be either positive everywhere or negative (cooling) along the top boundaries, as shown in Fig. 3f.

By incorporating heat diffusion equations for WTe_2_ and for the Si substrate into the numerical calculations (Methods), we can derive the corresponding δ*T*_J_, δ*T*_E_ and the total δ*T* distributions. Figure 3g–i shows that the calculated local temperatures follow the local $\dot{Q}$ maps in Fig. 3d–f, albeit broadened by the thermal heat conductivities in the flake and the substrate. In particular, the enhancement of ${\dot{Q}}_\mathrm{J}$ in the narrow slivers along the edges in Fig. 3d is completely broadened in the δ*T*_J_ map in Fig. 3g. We attain very good agreement between the numerical simulations and the experimental data for the microscopic parameters ${l}_{\mathrm{R}}^{0}=0.5\,\upmu\rm{m}$, *μ* = 25,000 cm^2^ V^−1^ s^−1^ and *ϵ*_p_ = 0.06 meV. The calculated temperature distributions shown in Fig. 3g–i match qualitatively very well the measured δ*T*_J_ and δ*T*_E_ maps in Fig. 1e,h and with the total δ*T* distribution in Fig. 2a. Figure 2c shows the calculated $\updelta T(y)$ profiles across the width of the sample at various currents, which reproduce well the experimental behaviour. Moreover, the experimental phase diagram of the absolute cooling $\updelta {T}_{\mathrm{cold}}(I,B)$ in Fig. 2e is also well described by the model (Fig. 2f), showing the accessible range of absolute cooling and the optimal parameters for achieving the lowest temperatures in our device. Finally, the model also recovers the non-monotonic width dependence and the nonlinearity of $\Delta {T}_\mathrm{E}\left(B\right)$ in Fig. 1l (solid lines).

## Analytical model

Further insight into the role of the different parameters in a mesoscopic device can be attained by considering a simplified model for the total heat fluxes. The lateral heat flux from the hot to the cold side of the device is governed by the in-plane total heat conductivity *κ* of WTe_2_ with thickness *d*. Further, heat flows from the hot side of the device to the substrate and from the substrate to the cold part of the device. The flow of heat depends on the out-of-plane heat conductivity *κ*′ of the substrate, which has effective thickness *d*_s_. In this geometry, we find (Methods) that the condition on the applied current for attaining a negative excess temperature at the cold edge is *I* < *I*_0_, where

6 $${I}_{0}\approx\frac{{\epsilon }_\mathrm{p}}{e{\rho }_{0}}\frac{4\mu B}{(1+{\mu }^{2}{B}^{2})\left(1+{W}_{\;0}^{\;2}/{W}^{\;2}\right)}$$

and ${W}_{0}\approx\sqrt{8d{d}_\mathrm{s}\kappa /{\kappa }^{{\prime} }}$. The dashed curve in Fig. 2e shows that the fit of *I*_0_(*B*) to the experimental data provides a very good description of the cooling regime. Note that from equation (6), the maximum of *I*_0_(*B*) occurs at *μB* = 1, which allows a direct evaluation of *μ* ≈ 23,000 cm^2^ V^−1^ s^−1^ from the experimental data, consistent with the more accurate 3D simulations, resulting in *μ* = 25,000 cm^2^ V^−1^ s^−1^.

By integrating ${\dot{Q}}_\mathrm{E}$ over the cold side of the sample and taking into account the above heat conductivities, we derive the transverse temperature difference (Methods):

7 $$\Delta {T}_\mathrm{E}\approx\frac{{{\epsilon }}_\mathrm{p}I}{ed\kappa }\frac{2\mu B}{(1+{\mu B{l}_\mathrm{R}^{0}}/{W}\,)\left(1+{{W}^{\,2}}/{{W}_{0}^{\,2}}\right)}.$$

This result emphasizes a number of key aspects of the mesoscopic nature of the Ettingshausen effect, which cannot be observed in bulk materials. The first is the sublinear dependence of Δ*T*_E_ on *B* due to the ${\mu B{l}_\mathrm{R}^{0}}/{W}$ term, which allows us to evaluate the electron–hole recombination length ${l}_\mathrm{R}^{0}\approx0.3\,\upmu\rm{m}$, which is close to the more accurate value derived numerically (Fig. 1l). In contrast, in the bulk limit of $W\gg \mu B{l}_\mathrm{R}^{0}$, the common linear *B* dependence of Δ*T*_E_ is recovered, so that we are unable to determine ${l}_{R}^{0}$ from such measurements.

**Extended Data Fig. 1:**
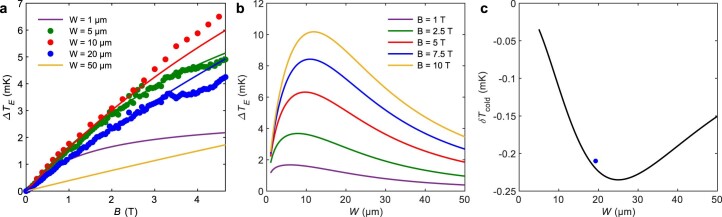
Analytical calculations of Ettingshausen effect and absolute cooling in mesoscopic devices. (**a**) Calculated Δ*T*_*E*_ vs. *B* using Equation 11 (solid lines), fitted to the experimental data from Fig. 1l for different widths *W*. The calculated Δ*T*_*E*_ curves show the nonmonotonic dependence on *W* and the sublinear *B* dependence for small *W*, which becomes linear for large *W*. The analytical curves, based on a simplified model, are slightly different from the numerical ones presented in Fig. 1l. (**b**) Calculated Δ*T*_*E*_ vs. *W* for different magnetic field values *B* showing nonmonotonic *W* dependence. The width *W* for which Δ*T*_*E*_ is largest grows monotonically with *B*. (**c**) Calculated $\delta {T}_{cold}^{\,max}\,$(black line) showing a nonmonotonic dependence on *W*, peaked at *W*_0_. The blue dot is the measured extremal $\delta {T}_{cold}$ from Fig. 2e. Source data

Second, equation (7) predicts a non-monotonic dependence of Δ*T*_E_ on *W*, which describes well the behaviour in Fig. 1l (see Extended Data Fig. 1 for more details). The largest Δ*T*_E_ should be attainable for $W\approx\sqrt[3]{{W}_{\;0}^{\;2}{l}_{\,\mathrm{R}}^{0}}\mu B$ for *μB* < 1. From our observation of the largest transverse temperature difference in the *W* *=* 10 µm sample, we derive *W*_0_ ≈ 24 μm. This new mesoscopic length scale *W*_0_ arises due to the competition between the transverse heat conductivity across the sample width and the heat conductivity to the substrate and is absent in the bulk case. This length scale can serve as an important parameter for further optimization of Ettingshausen cooling in microscopic devices.

Third, we derive that the largest cooling is attained at $\hat{I}={I}_{0}/2$ and $\hat{B}=\frac{1}{\mu }\sqrt[3]{\frac{2W}{{l}_\mathrm{R}^{0}}}$ (Methods) where the negative excess temperature reaches

8 $$\updelta {T}_\mathrm{cold}^{\,\max}\approx-\frac{{{\epsilon }}_\mathrm{p}^{2}}{{e}^{2}{\rho }_{0}d\kappa }\frac{{W}_{0}^{\;2}{W}^{\;2}}{{\left({W}^{\,2}+{W}_{0}^{\,2}\right)}^{2}\,}.$$

For *W* = 20 μm, this results in $\hat{B}\approx2.2\,{\rm{T}}$ and ${\updelta T}_{\mathrm{cold}}^{\,\max }\approx-0.2\,{\rm{mK}}$, in good agreement with Fig. 2d.

Equation (8) shows that lower cooling temperatures can be attained by reducing the sample sheet resistivity *ρ*_0_. In our WTe_2_ flake, *ρ*_0_ ≈ 0.54 Ω, corresponding to an effective bulk resistivity *ρ*_0_*d* ≈ 1.5 × 10^−5^ Ω cm (*d* = 269 nm), which is almost two orders of magnitude higher than the low-temperature bulk resistivity *ρ* ≈ 2.3 × 10^−7^ Ω cm in high-quality crystals^31^. Since the increase in *ρ*_0_ is attributed to surface oxidation^54^, one can expect to attain substantially stronger cooling in devices fabricated in an inert environment and encapsulated in hBN.

Finally, from the combination of the derived parameters (Methods), we extracted the Ettingshausen conversion coefficient *ϵ*_p_ ≈ 0.06 meV, which is about one sixth of the thermal energy of the quasiparticles (*k*_B_*T*_0_ = 0.37 meV) and, thus, in good agreement with the description of the Ettingshausen effect in terms of particle–hole generation and recombination.

The developed nanoscale imaging of magneto-electro-thermal cooling at cryogenic temperatures in microscopic devices opens the door for studying and engineering thermal landscapes and energy harvesting in new electronic materials and devices. The derived microscopic model allows one to further enhance the cooling efficiency through the optimization of the electrical and thermal conductivity characteristics of the materials and of the device geometries. This study suggests the possibility of integrating transition metal dichalcogenide atomic layer semimetals in multilayer vdW heterostructures to provide for in-device electro-thermal cooling, temperature gradient engineering and studying thermoelectric effects in low-dimensional strongly correlated states of matter.

## Methods

### WTe_2_ device fabrication

High-quality WTe_2_ single crystals were grown using a flux growth technique^51^ as described in ref. ^31^. Bulk samples produced by this method showed a residual resistance ratio of 3,250, a magnetoresistance ratio of up to 62,000 at 9 T and 2 K, and a mean free path *l*_mr_ ≈ 20 µm (ref. ^31^). The crystals were then used to mechanically exfoliate WTe_2_ flakes onto oxidized silicon wafers (290 nm of SiO_2_). Suitable flakes, identified by optical and atomic force microscopy, were then processed by electron-beam lithography and inductively coupled plasma etching into the various geometries. The latter was done with a flow of 20 sccm of SF_6_ and 10 sccm of O_2_ at a radio-frequency power of 25 W, which provides an etching rate of WTe_2_ of about 4.7 nm min^−1^. Electrical contacts were fabricated by an additional electron-beam lithography step, Ar ion milling to remove the native oxide layer of WTe_2_, followed by electron-beam deposition of 3 nm of Ti and Au to a thickness that exceeded the WTe_2_ flake thickness. Transport measurements of the flakes show carrier mobilities of about 10,000 to 30,000 cm^2^ V^−1^ s^−1^ at 4 K, corresponding to a mean free path of *l*_mr_ ≈ 0.7 to 2 µm, more than an order of magnitude lower than the bulk values due to surface oxidation and scattering, as reported previously^31,54^.

### SOT fabrication and thermal imaging

Nanoscale SOTs have been used as high-precision local thermometers to study dissipation in mesoscopic systems^50,55–57^. This is achieved through the strong dependence of the SOT critical current ${I}_{\mathrm{c}}(T\;)$ on the SOT temperature. In practice, the SOT is connected in parallel with a small shunt resistor and biased above the critical current, ${I}_{\mathrm{bias}} > {I}_\mathrm{c}$. As a result, the current through the SOT ${I}_{\mathrm{SOT}}(T\;)$ has a temperature dependence that approximately follows ${I}_{\mathrm{c}}(T\;)$, with the best sensitivity in the temperature range ${T}_\mathrm{c}/2 < T < {T}_\mathrm{c}$. The thermal coupling between the SOT and the sample was achieved with a low-pressure He exchange gas. A cryogenic SQUID series array amplifier^58^ was used for the *I*_SOT_ readout.

SOTs with a diameter around 110 nm were fabricated as described in refs. ^48,59^. In this work, MoRe (*T*_c_ ≈ 7.2 K) was used as the superconducting material for the SOT to provide thermal sensitivity throughout the magnetic field range up to 5 T (ref. ^49^) with a thermal sensitivity at zero field of 2.6 µK Hz^−^^1/2^. Calibrating the temperature sensitivity of the SOT at zero field was carried out using a heater installed in the microscope. The relative magnetic field dependence of the sensitivity was derived by imaging the temperature of an Au thin-film heater, which was patterned next to the WTe_2_ flake, versus *B*. The MoRe SOT was intentionally chosen to have a weak sensitivity to a magnetic field that decreased rapidly with the magnetic field^49^. All data presented were acquired when the magnetic signal due to the Oersted field of the current flowing in the sample was negligible compared with the thermal signal, except for Fig. 1g, where a small signal at zero field can be resolved in the constrictions.

The SOT height was controlled by attaching the tip to a quartz tuning fork^50^, which allowed scanning at a height of 80 nm above the sample surface. Thermal imaging was done at a base temperature of 4.3 K and in an environment of 40 mbar of He exchange gas to provide the thermal link between the tip and the sample^50^. All images in the main text were acquired with image resolution of 344 × 269 pixels, a pixel size of 90 nm and an acquisition time of 40 ms per pixel.

For the scans in Fig. 1, an a.c. bipolar sine wave with an rms current of *I* = 50 μA and an excitation frequency of *f* = 85.37 Hz was applied, and the resulting thermal response of the SOT was measured at the first and second harmonics of *f* using a lock-in amplifier. Note that a current $I\left(t\right)={I}_{0}\cos (\omega t)$ creates Joule heating ${\dot{Q}}_\mathrm{J}(t)=R{I}^{2}(t)=\frac{1}{2}R{I}_{0}^{2}+\frac{1}{2}R{I}_{0}^{2}\cos (2\omega t)$, which has a d.c. component and a superimposed a.c. component at twice the excitation frequency. The latter a.c. component was measured by the second harmonic signal of the SOT.

To capture the total current-induced local temperature change in the sample δ*T* relative to the base temperature *T*_0_, we applied a unipolar square-wave excitation and measured the difference between the current-on and the current-off states, as presented in Fig. 2a. Negative δ*T* means absolute current-induced cooling. That is, in the current-on state, the local temperature is lower than in the current-off state.

### Analytical derivation of the Ettingshausen effect

Here we describe the simplified heat flux model. The results are in good agreement with the full numerical simulations and provide deeper insights into the role of the different parameters. We assume an approximately linear temperature gradient across the width $-{W}/{2}\le y\le {W}/{2}$ of the sample, $T\left(\;y\right)={T}_{0}-\left|\updelta {T}_{\mathrm{cold}}\right|+(0.5+y/W\;)\,\Delta {T}_\mathrm{E}$, and calculate the lateral and out-of-plane heat fluxes. The lateral heat flux through the flake from the hot to the cold side of the device is ${q}_{0}=d\kappa \Delta {T}_\mathrm{E}/W$. We compare the heat fluxes into and out of the substrate by separately considering the two regions of the sample $-{W}/{2}\le y\le 0$ and $0 < y\le {W}/{2}$. By integrating over each region, we attain the heat flux from the hotter part of the sample into the substrate:

$${q}_\mathrm{h}=\tfrac{{\kappa }^{{\prime} }}{{d}_\mathrm{s}}\int_{0}^{W/2}(T\left(\,y\right)-{T}_{0})\,\mathrm{d}y=W\kappa^{\prime} (3\Delta {T}_\mathrm{E}-4|\updelta {T}_{\mathrm{cold}}|)/8{d}_\mathrm{s}.$$

Similarly, the heat flux from the substrate into the colder side:

$${q}_\mathrm{c}=\frac{{\kappa }^{{\prime} }}{{d}_\mathrm{s}}\int_{-W/2}^{0}({T}_{0}-T(\,y))\,\mathrm{d}y=W\kappa^{\prime} (4\left|{\updelta T}_{\mathrm{cold}}\right|-\Delta {T}_\mathrm{E})/8{d}_\mathrm{s}.$$

This leads to ${q}_\mathrm{h}+{q}_\mathrm{c}=W{\kappa }^{{\prime} }\Delta {T}_\mathrm{E}/4{d}_\mathrm{s}$ and ${q}_\mathrm{h}-{q}_\mathrm{c}=W\kappa^{\prime} (\Delta {T}_\mathrm{E}-2|{\updelta T}_{\mathrm{cold}}|)/2{d}_\mathrm{s}$. The total heat flow generated by the Ettingshausen heating is, thus, described by

9 $$2\int_{-W/2}^{0}{\dot{Q}}_\mathrm{E}\,{\rm{d}}y\approx2{q}_{0}+{q}_\mathrm{h}+{q}_\mathrm{c}=\left(\frac{2d\kappa }{W}+\frac{W\kappa^{\prime} }{4{d}_\mathrm{s}}\right)\Delta {T}_\mathrm{E}.$$

The heat flow due to Joule heating in the same area is given by:

10 $$2\int_{-W/2}^{0}{\dot{Q}}_\mathrm{J}\,{\rm{d}}y={q}_\mathrm{h}-{q}_\mathrm{c}=\frac{W\kappa^{\prime} }{2{d}_\mathrm{s}}\left(\Delta {T}_\mathrm{E}-2|\updelta {T}_\mathrm{cold}|\right).$$

Introducing the length scale ${W}_{0}=\sqrt{8d{d}_\mathrm{s}{\kappa}/{{\kappa}^{{\prime}}}}$ and solving for $|\updelta {T}_{\mathrm{cold}}|$ and the temperature difference Δ*T*_E_, thus results in

11 $$\Delta{T}_\mathrm{E}=\frac{{{\epsilon}}_\mathrm{p}I}{ed\kappa}\frac{2\mu B}{\left(1+\mu B{l}_\mathrm{R}^{0}/W\,\right)\left(1+{W}^{\;2}/{W}_{0}^{2}\right)},$$

12 $$|\updelta {T}_\mathrm{cold}|=\frac{\frac{{\rho }_{0}{I}^{2}}{4}\left(1+{\mu }^{2}{B}^{2}\right)-I\frac{{{\epsilon }}_\mathrm{p}}{e}\frac{\mu B}{1+{W}_{0}^{\,2}/{W}^{\,2}}}{d\kappa \frac{{W}^{\,2}}{{W}_{0}^{\,2}}\left(1+\frac{{\mu }^{2}{B}^{2}}{\sqrt{1+{\mu }^{2}{B}^{2}}}\frac{{l}_\mathrm{R}^{0}}{W}\right)}.$$

The current at which the Joule heating at the cold edge balances the Ettingshausen cooling, resulting in $\left|{\updelta T}_{\mathrm{cold}}\right|=0$, is thus given by

13 $${I}_{0}\approx\frac{{{\epsilon }}_\mathrm{p}}{e{\rho }_{0}}\frac{4\mu B}{(1+{\mu }^{2}{B}^{2})\left(1+\frac{{W}_{0}^{\,2}}{{W}^{\,2}}\right)}.$$

The maximal cooling is attained at $\hat{I}={I}_{0}/2$ and $\hat{B}=\frac{1}{\mu }\sqrt[3]{\frac{2W}{{l}_\mathrm{R}^{0}}}$, with the lowest temperature of

14 $$\updelta {T}_\mathrm{cold}^{\,\max}(\hat{B})\approx-\frac{{{\epsilon }}_\mathrm{p}^{2}}{{e}^{2}{\rho }_{0}d\kappa }\frac{{W}_{0}^{\;2}{W}^{\,2}}{{\left({W}^{\,2}+{W}_{0}^{\;2}\right)}^{2}\,}.$$

The fit to the experimental data is performed in three steps. The values of *μ* and the combined coefficient ${C}_{1}=\frac{{\epsilon }_\mathrm{p}}{e{\rho }_{0}}\frac{1}{(1+{W}_{0}^{2}/{W}^{2})}$ can be determined from fitting the line $\left|{\updelta T}_{\mathrm{cold}}\right|=0$ in the phase diagram in Fig. 2e. Subsequently, the non-monotonic dependence of Δ*T*_E_ as a function of *W* for a given current *I* in equation (11) can be used to yield estimates for *W*_0_, ${l}_\mathrm{R}^{0}$ and the global coefficient ${C}_{2}=\frac{{{\epsilon}}_\mathrm{p}}{ed\kappa}$, as shown in Extended Data Fig. 1a,b. These values can then be used to evaluate ${\updelta T}_{\mathrm{cold}}^{\,\max }=-{C}_{1}{C}_{2}\frac{{W}_{\,0}^{\;2}}{\left({W}^{\;2}+{W}_{\,0}^{\;2}\right)\,}$ (Extended Data Fig. 1c). Finally, from *C*_1_ and using the zero-field sheet resistance *ρ*_0_ ≈ 0.54 Ω derived from the full numerical fits, we can evaluate *ϵ*_p_ ≈ 0.06 meV.

Figure 2e,f and Extended Data Fig. 1 show that the analytical derivation captures well the essence of the Ettingshausen effect and of the absolute cooling in mesoscopic devices, although it considers a simplified model compared with the numerical simulations presented in Figs. 1l and 2f. The fitted values of *μ* and ${l}_\mathrm{R}^{0}$ are slightly different between the analytical fits and the numerical simulations.

### Numerical simulations

Finite-element 3D numerical simulations of the temperature maps due to Joule heating and the Ettingshausen effect in a nearly compensated semimetal were used to fit the experimental data. The calculations were conducted using COMSOL Multiphysics 5.4 in two consecutive steps. In the first step, the transport equations of a two-component conductor^53^ were solved:

15a $$\frac{{l}_{\mathrm{R}}^{\;2}}{{\tau }_\mathrm{R}}\nabla \delta {n}_{\alpha }-{n}_{\alpha }{\mu }_{\alpha }{{{\mathbf{E}}}}-{{{{\mathbf{j}}}}}_{\alpha }\times (\;{\mu }_{\alpha }{{{\mathbf{B}}}})=-{{{{\mathbf{j}}}}}_{\alpha }$$

15b $$\operatorname{div}{{\bf{j}}}_\mathrm{e(h)}=-\frac{\delta {n}_\mathrm{q}}{2{\tau }_\mathrm{R}},$$

where the index *α* *=* e or h describes electrons or holes, **j**_*α*_ are the particle flux densities, ${{{\mathbf{E}}}}=-\nabla \phi$ is the electric field, *ϕ* is the electric potential, *n*_e_ and *n*_h_ are the equilibrium densities of electrons and holes, $\updelta {n}_{\mathrm{q}}=\updelta {n}_{\mathrm{e}}+\updelta {n}_{\mathrm{h}}$ is the total out-of-equilibrium quasiparticle density, ${\mu }_{{\mathrm{e}}\left({\mathrm{h}}\right)}$ is the electron (hole) mobility, *τ*_R_ is the characteristic recombination time and *l*_R_ is the recombination length. The recombination length and time are related by ${l}_{\mathrm{R}}=\sqrt{D{\tau }_{\mathrm{R}}}$, where *D* is the diffusion coefficient. To represent these equations, we used the Coefficients Form PDE module, which solves the general equation:

16 $${e}_{a}\frac{{\partial }^{2}{{{\mathbf{u}}}}}{\partial {t}^{2}}+{d}_{a}\frac{\partial {\mathbf{u}}}{\partial t}+\nabla \cdot (-c\nabla {{{\mathbf{u}}}}-\alpha {{{\mathbf{u}}}}+\gamma )+\beta \cdot \nabla {{{\mathbf{u}}}}+a{{{\mathbf{u}}}}=f,$$

where the field **u** is

17 $${{{\mathbf{u}}}}=\begin{pmatrix}\tilde{\phi}&\widetilde{\updelta{n}_\mathrm{q}}&e{j}_{\mathrm{e},x}&e{j}_{\mathrm{e},y}&e{j}_{\mathrm{h},x}&e{j}_{\mathrm{h},y}\end{pmatrix}.$$

Here $\widetilde{\phi }=\left(\frac{{e}^{2}}{h}\right)\frac{1}{{ld}}\phi$, $\widetilde{\updelta {n}_\mathrm{q}}=({{l}_\mathrm{R}}/{{\tau }_\mathrm{R}})\;e\updelta {n}_\mathrm{q}$ and *l* = 1 μm is a length scale for expressing ***u*** in units of current density. Equation (16) was solved in a 3D geometry that follows the dimensions of the experimental sample depicted in Fig. 1c, using fitted parameters *μ*_e_ = *μ*_h_ = 25,000 cm^2^ V^−1^ s^−1^, *n*_e_ = *n*_h_ = 2.1 × 10^18^ cm^−3^ and ${l}_\mathrm{R}^{0}=0.5\,\upmu\rm{m}$.

The heat transport was solved in the second step of the simulation. For this, we employed a heat transfer in solids module to simulate heat diffusion in the system. The 3D geometry consisted of the WTe_2_ sample of thickness *d* = 269 nm, heat conductivity of ${\kappa }_{\text{WT}{\text{e}}_{2}}=6.4\,{\rm{W}}\,{\rm{K}}^{-1}\,{\rm{m}}^{-1}$, a He exchange gas layer surrounding the sample that extends 1 μm above it (*κ*_He_ = 0.005 W K^−^^1^ m^−^^1^), a SiO_2_ layer of thickness 300 nm (${\kappa}_{\text{Si}{\text{O}}_{2}}=0.2\,{\rm{W}}\,{\rm{K}}^{-1}\,{\rm{m}}^{-1}$), and a Si layer of thickness 5 μm (*κ*_Si_ = 0.32 W K^−^^1^ m^−^^1^), which was applied for practical convenience. From the solution of equation (16) in the first step, we calculated ${\dot{Q}}_\mathrm{E}={\epsilon}_\mathrm{p}\updelta{n}_\mathrm{q}/{\tau}_\mathrm{R}$, ${\dot{Q}}_\mathrm{J}=-{\mathbf{J}}\cdot \nabla \phi$, and $\dot{Q}={\dot{Q}}_\mathrm{J}+{\dot{Q}}_\mathrm{E}$ with *ϵ*_p_ = 0.16*k*_b_*T*_0_ = 0.06 meV, and used these as the heat sources for the heat transfer module to solve for δ*T*_E_, δ*T*_J_ and δ*T*. The bottom side of the Si layer was set as the thermal anchor to *T*_0_ = 4.3 K.

Note that although ${l}_\mathrm{R}^{0}$ and *μ* affect the fitting to the experimental data substantially, the rest of the parameters provide a fit over a range of values. The simulations presented should, thus, be considered as a qualitative demonstration of the validity of the model rather than an accurate determination of the parameter values.

### Ettingshausen effect in additional samples

#### Strip geometry

**Extended Data Fig. 2:**
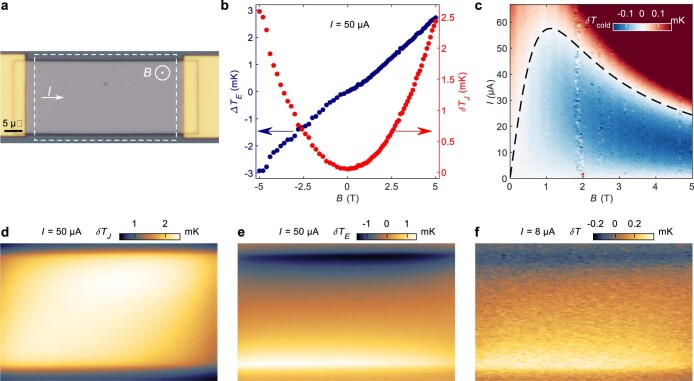
Thermal imaging and absolute cooling in a sample with strip geometry. (**a**) Optical image of the sample with length *L* = 42 μm and width *W* = 20 μm with Au contacts on the left and right sides. (**b**) Temperature difference *∆T*_*E*_ between the bottom and the top edge (blue curve) and *δT*_*J*_ in the center of the strip (red) vs. *B* at *ac* current of 50 µA. (**c**) The temperature of the cold edge, *δT*_*cold*_, as a function of *B* and the applied unipolar current *I*. Dashed line shows a fit to the analytically calculated ${I}_{0}(B)$. (**d**) Thermal map of *δT*_*J*_ at *B* = 5T and *I* = 50 µA. (**e**) Corresponding map of *δT*_*E*_. (**f**) Map of the total excess temperature *δT* using unipolar square wave current *I* = 8 μA at *B* = 5T, revealing absolute cooling at the top edge. Source data

To exclude excess heating arising from the narrow constrictions in the rectangular chamber geometry, a sample with a strip geometry (Extended Data Fig. 2a) of width *W* = 20 µm and thickness *d* = 160 nm was investigated. By applying a sinusoidal a.c. current with *I* = 50 µA at *B* = 5 T, we acquired the maps of δ*T*_J_ and δ*T*_E_ shown in Extended Data Fig. 2d,e. The absence of the constrictions resulted in more uniform temperature distributions in the device compared with Fig. 1f,i. The slight canting of the thermal distributions in Extended Data Fig. 2d,e is probably due to the small Hall voltage resulting from incomplete compensation between the electron and hole densities and mobilities and shows an opposite tilt angle upon reversing the magnetic field direction (Supplementary Information). A quantitative analysis of the field dependence of δ*T*_J_ at the centre of the device (Extended Data Fig. 2b) shows quadratic behaviour that approximately follows the *B* dependence of the sample resistivity, whereas the temperature difference ${\Delta T}_\mathrm{E}={\updelta T}_\mathrm{E}\left(0,-{W}/{2}\right)-\updelta {T}_\mathrm{E}\left(0,{W}/{2}\right)$ between the hot and cold edges across the centre of the strip shows an approximately linear *B* dependence consistent with Fig. 1k,l.

Applying a unipolar square-wave excitation with *I* = 8 µA at *B* = 5 T reveals absolute cooling at the top edge in Extended Data Fig. 2f. The phase space for absolute cooling was found by mapping δ*T*_cold_ versus *B* and *I*, as shown in Extended Data Fig. 2c. The overall behaviour is like the *W* = 20 µm device in Fig. 2e with comparable δ*T*_cold_ values. The main two quantitative differences, however, are the position of the peak of ${I}_{0}(B)$, which occurs at *B* ≈ 1.1 T in Extended Data Fig. 2c, and the maximal negative $\updelta {T}_{{{\mathrm{cold}}}}^{\max }$ attained at $B\gtrsim \, 5$ T. By fitting the ${I}_{0}\left(B\right)$ (dashed line in Extended Data Fig. 2c), we find *μ* = 9,000 cm^2^ V^−1^ s^−1^. This lower value is consistent with this device being thinner^31,54^.

#### Rectangular geometry

**Extended Data Fig. 3:**
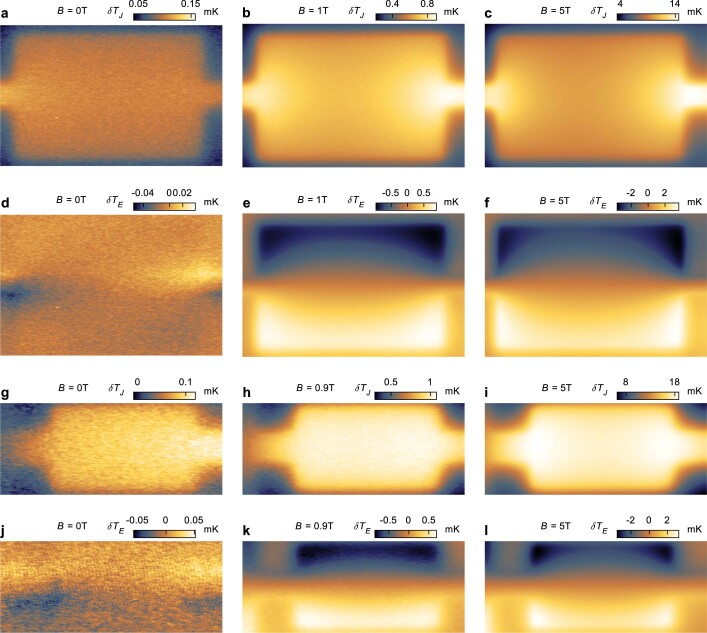
Thermal imaging of additional chambers in rectangular geometry. (**a-c**) Maps of *δT*_*J*_ at different indicated magnetic field values for the rectangular chamber with width *W* = 10 μm at *I* = 50 μA. (d-f) Corresponding maps of *δT*_*E*_. (**g-i**) Maps of *δT*_*J*_ for the chamber with width *W* = 5 μm. (**j-l**) Corresponding maps of *δT*_*E*_.

We present here thermal imaging of an additional two chambers of width *W* = 10 and 5 µm connected in series with the *W* = 20 µm chamber described in the main text (Fig. 1c). Extended Data Fig. 3a–c shows δ*T*_J_ maps at *B* = 0, 1 and 5 T in the *W* = 10 µm chamber at *I* = 50 µA with corresponding δ*T*_E_ maps in Extended Data Fig. 3d–f. A similar set of data is presented in Extended Data Fig. 3g–l for the *W* = 5 µm chamber. The qualitative behaviour is like that of the *W* = 20 µm chamber. However, *∆T*_E_ shows a non-monotonic dependence on *W*, as described in Fig. 1l.

#### Additional geometries

**Extended Data Fig. 4:**
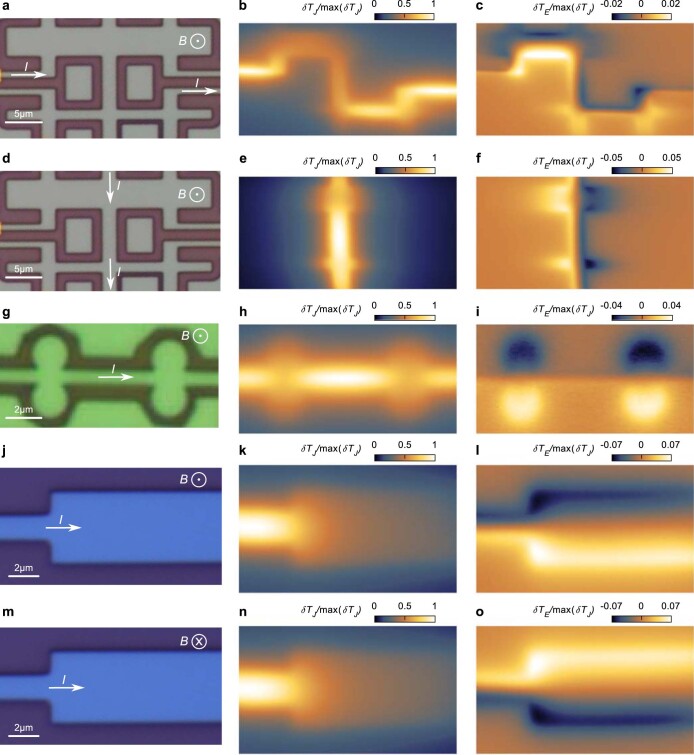
Thermal imaging in additional geometries. (**a**) Optical image of a WTe_2_ sample with strips of different width (grey). The width of the top horizontal strip is *W* = 4 μm and of the lower strip is *W* = 1 μm. The white arrows show the current source and drain. (**b**) Thermal map of *δT*_*J*_ normalized by its maximal value $\max (\delta {T}_{J})$ for the current flow configuration in (a). (**c**) The corresponding thermal map of *δT*_*E*_ normalized by the same value as in (b). (**d**) Same as a, but with a current configuration along the central vertical strip (white arrows). (**e**, **f**) Thermal maps of $\delta {T}_{J}/\,\max (\delta {T}_{J})$ and $\delta {T}_{E}/\,\max (\delta {T}_{J})$ respectively for the current configuration in (d). (**g**) Optical image of a WTe_2_ sample with a narrow strip of width *W* = 0.55 µm connected to circular chambers. (**h**, **i**) $\delta {T}_{J}/\,\max (\delta {T}_{J})$ and $\delta {T}_{E}/\,\max (\delta {T}_{J})$ for the current configuration in (g). (**j**) Optical image of a WTe_2_ sample with two regions of different width in series. The magnetic field ***B*** points out of the plane. (**k**, **l**) $\delta {T}_{J}/\,\max (\delta {T}_{J})$ and $\delta {T}_{E}/\,\max (\delta {T}_{J})$ for the configuration in (J). (**m**) Same as (j) but with ***B*** pointing in the opposite direction. (**n**) $\delta {T}_{J}/\,\max (\delta {T}_{J})$ for the configuration in (m), showing the same distribution as in (k). (**o**) $\delta {T}_{E}/\,\max (\delta {T}_{J})$ for the configuration in m, showing an opposite temperature gradient relative to (l) due to the inversion of ***B***.

Additional samples with more complex geometries are presented in Extended Data Fig. 4. Since an accurate calibration of the SOT thermal response was not available in this set of experiments, the data are presented in relative units.

Extended Data Fig. 4a,d shows a sample with thickness *d* = 110 nm, which allows the application of current through channels of different widths and along different directions. For the current path shown in Extended Data Fig. 4a, the largest δ*T*_J_ was observed in Extended Data Fig. 4b in the two narrowest channels on the left and right sides. In contrast, the largest transverse *∆T*_E_ occurs in the widest top left channel in Extended Data Fig. 4c. Additionally, strongly non-local Ettingshausen heating and cooling can be observed in the vicinity of the right-angled corners along the current path, as discussed below.

Application of the current along the central vertical channel in Extended Data Fig. 4d reveals that *∆T*_E_ in the vertical strip sections in Extended Data Fig. 4f is smaller than the Ettingshausen heating and cooling spots appearing at the junctions with the horizontal channels. The temperature maps for the two different current paths in the sample illustrate the mesoscopic nature of the Ettingshausen effect, as discussed in the main text, along with its non-local character. The Joule heating is local in nature because ${\dot{Q}}_\mathrm{J}$ is mainly determined by the local *J*. In contrast, ${\dot{Q}}_\mathrm{E}$ is not determined by the local *P* but rather by $\nabla \cdot {{{\mathbf{P}}}}$.

These features were further corroborated by thermal imaging of the sample shown in Extended Data Fig. 4g, which has thickness *d* = 48 nm and was used in ref. ^31^. This sample consists of a very narrow strip of *W* = 0.55 µm connected to two circular discs of 1.8 µm diameter. Extended Data Fig. 4h,i shows that δ*T*_J_ is largest along the narrow horizontal strip, whereas ∆*T*_E_ is vanishingly small. In narrow channels, with width comparable to *l*_R_, the regions of electron–hole generation and recombination partially overlap and the induced Ettingshausen temperature gradient is greatly suppressed by the large transverse heat conductivity across the narrow channel. In sharp contrast, Extended Data Fig. 4i shows very pronounced Ettingshausen heating and cooling in the circular chambers, where almost no current flows, emphasizing its non-local character.

Qualitatively, the dependence of δ*T*_E_ on the channel width can also be seen in the temperature maps of the sample depicted in Extended Data Fig. 4j,m, which has two connected channels of different widths and a thickness of *d* = 105 nm. As seen in Extended Data Fig. 4l, the temperature difference between the hot and cold edges *∆T*_E_ is larger in the wider channel and there is a pronounced increase in Ettingshausen heating and cooling at the corners, like the results in the main text (Fig. 1h,i). As expected for the Ettingshausen effect, δ*T*_E_ reverses sign upon reversing the magnetic field direction in Extended Data Fig. 4o, whereas the Joule heating shown in Extended Data Fig. 4k,n remains unaffected.

## Online content

Any methods, additional references, Nature Portfolio reporting summaries, source data, extended data, supplementary information, acknowledgements, peer review information; details of author contributions and competing interests; and statements of data and code availability are available at 10.1038/s41567-024-02417-z.

### Supplementary information

Supplementary Information Supplementary Fig. 1 and a discussion of Supplementary Fig. 1.

### Source data

Source Data for Fig. 1 Source data for Fig. 1j–l.

Source Data for Fig. 2 Source data for Fig. 2b–d.

Source Data for Extended Data Fig. 1 Source data for Extended Data Fig. 1a–c.

Source Data for Extended Data Fig. 2 Source data for Extended Data Fig. 2b.

## Data Availability

The data that support the findings of this study are available from the corresponding author on reasonable request.

## Extended data

Extended data are available for this paper at 10.1038/s41567-024-02417-z.

## Code availability

The finite-element COMSOL numerical simulation codes are available from the corresponding author on reasonable request.
