# Supplementary material for: Demonstration and imaging of cryogenic magneto-thermoelectric cooling in a van der Waals semimetal
Source: Nat Phys. 2024 Mar 8;20(6):976–83. doi: 10.1038/s41567-024-02417-z (PMC11178502; doi:10.1038/s41567-024-02417-z)
Supplement: Supplementary file 1 — Supplementary Fig. 1 and a discussion of Supplementary Fig. 1. [file 41567_2024_2417_MOESM1_ESM.pdf]

nature physics

Article https://doi.org/10.1038/s41567-024-02417-z

# Demonstration and imaging of cryogenic magneto-thermoelectric cooling in a van der Waals semimetal

In the format provided by the authors and unedited

## Supplementary Information

Here, we show the effect of reversing the magnetic field direction on the thermal measurements. Figure S1 displays thermal measurements of the sample discussed in Extended Data Fig. 2. As can be seen, changing the magnetic field from 5 T to −5 T results in reversal of the sign of $\delta T_E$ in Figs. S1b and S1d as expected. Further, the small canting of the thermal distribution seen for $\delta T_J$ in Fig. S1a is also reversed when reversing the magnetic field in Fig. S1c. Therefore, it can be assumed that this canting is caused by a small Hall-voltage as a result of the not perfect compensation between electrons and holes.

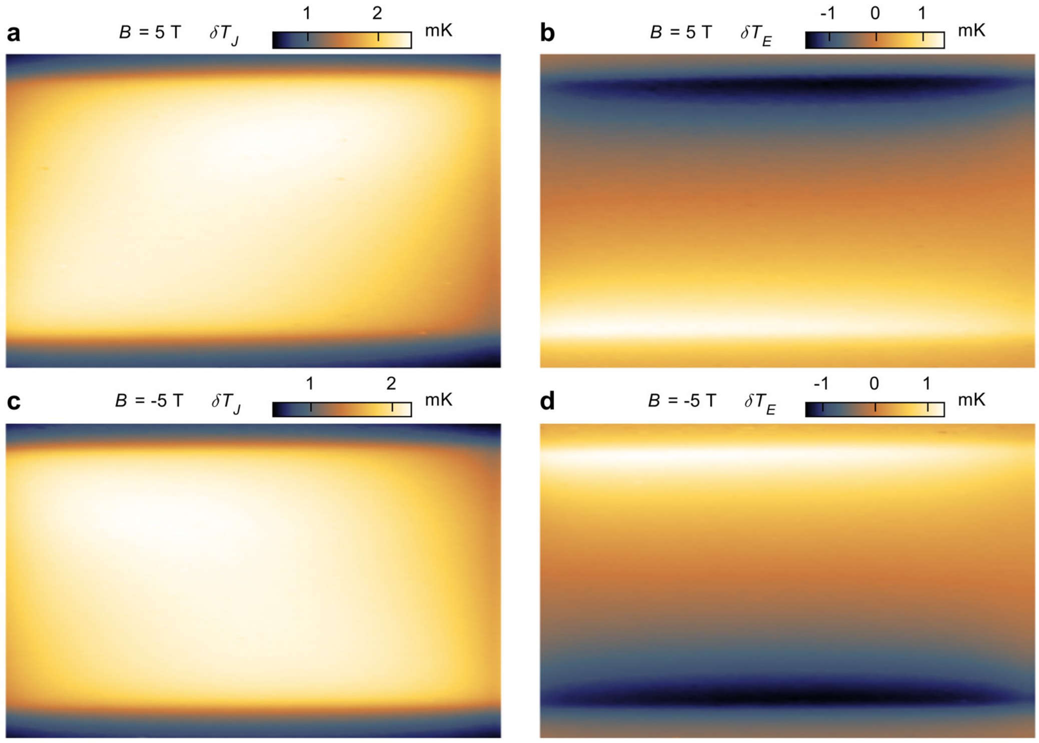


**Fig. S1. Effect of magnetic field reversal.** (**a-d**) Reversal of the magnetic field direction reverses the sign of $\delta T_E$ from (**b**) to (**d**), as well as the direction of the canting in $\delta T_J$ from (**a**) to (**c**).
